# First preimplantation genetic testing case of Meckel syndrome with a novel homozygous *TXNDC15* variant in a non‐consanguineous Chinese family

**DOI:** 10.1002/mgg3.2340

**Published:** 2023-12-11

**Authors:** Huiling Xu, Jiajie Pu, Ningjie Yang, Zhengzhong Wu, Chanlin Han, Jilong Yao, Xuemei Li

**Affiliations:** ^1^ Department of Reproductive Medicine Shenzhen Maternity & Child Healthcare Hospital Shenzhen Guangdong China; ^2^ Department of Bioinformatics 01life Institute Shenzhen Guangdong China

**Keywords:** Meckel–Gruber syndrome, novel variant, PGT‐M, trio‐WES, *TXNDC15*

## Abstract

**Background:**

Meckel–Gruber syndrome (MKS) is a perinatally lethal, genetically heterogeneous, autosomal recessive condition caused by defective primary cilium formation. So far, the association of *TXNDC15*‐related MKS has been reported in only five independent families from diverse ethnic origins, including Saudi, Pakistani, Estonian, and Indian. Here, we report a fetus diagnosed with MKS at 12 weeks, exhibiting typical ultrasound findings.

**Methods:**

Low‐coverage whole‐genome sequencing was used to identify chromosomal abnormalities. Trio‐base whole exome sequencing (trio‐WES) was performed to investigate the potential pathogenic variants associated with MKS. Preimplantation genetic testing for monogenic disorders (PGT‐M) was applied to prevent the transmission of the pathogenic variant.

**Results:**

A novel homozygous pathogenic variant in the *TXNDC15* gene was identified through trio‐WES. The application of PGT‐M successfully prevented the transmission of the pathogenic variant and resulted in an ongoing pregnancy.

**Conclusion:**

This is the first report of a *TXNDC15* variant in the Chinese population and the first PGT case of *TXNDC15*‐related MKS worldwide. The successful application of PGT‐M in this family provides a potential approach for other monogenic diseases. Our case expands the variant spectrum of *TXNDC15* and contributes to the molecular diagnosis and genetic counseling for MKS. This case underscores the importance of appropriate genetic testing methods and accurate genetic counseling in the diagnosis of rare monogenic diseases.

## Graphical abstract

This manuscript reports a fetus from a non‐consanguineous Chinese family with Meckel–Gruber syndrome (MKS) caused by a novel homozygous *TXNDC15* gene variant. Prenatal and preimplantation genetic diagnosis were applied to prevent the transmission of this pathogenic variant. It is the first report of pathogenic *TXNDC15* variant in Chinese population and the first preimplantation genetic testing case of *TXNDC15*‐related MKS worldwide. We summarized the *TXNDC15* variants and the corresponding patients' clinical data of 5 previously reported cases as well as our case.

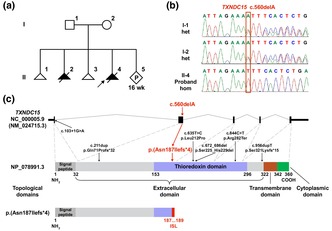

## 1 INTRODUCTION

The primary cilium is an essential sensory and signaling organelle protruding from the apical surface of almost all cell types in the human body (Shaheen et al., 2016; Ware et al., 2011; Wheway et al., 2018). Meckel–Gruber syndrome (MKS), which is a severe multiorgan dysplastic lethal ciliopathy with extreme genetic heterogeneity, is one of the developmental disorders caused by mutations affecting cilia (Breslow et al., 2018; Zhang et al., 2022). MKS was first described by Johann Friedrich Meckel in 1822 (Hartill et al., 2017). It can be detected on prenatal ultrasound in the first trimester and is usually characterized by encephalocele, postaxial polydactyly of the hands and feet, and polycystic dysplastic kidneys (Zhang et al., 2020). The global incidence of MKS ranges from 1 per 1300 in Gujarati Indians to 1 per 140,000 in England, while its prevalence in China remains unclear (Barisic et al., 2015; Zhang et al., 2020). At least 21 genes are known to cause MKS (Zhang et al., 2020). Biallelic variants of *TXNDC15* gene (OMIM: * 617778) which encodes a thioredoxin‐domain containing transmembrane protein are the cause of Meckel syndrome 14 (MKS14, OMIM: # 619879) (Breslow et al., 2018). As of the preparation of this manuscript, *TXNDC15*‐related MKS have only been reported in five independent families, including the first report supporting *TXNDC15* as a novel causative gene of MKS (Radhakrishnan et al., 2019; Ridnõi et al., 2019; Shaheen et al., 2016).

Here, we detail the case of a Chinese family with a recurrent history of adverse pregnancies due to MKS, caused by a novel homozygous pathogenic variant in the *TXNDC15* gene. This is the first recorded case of MKS14 in the Chinese population. The couple received genetic counseling and opted for preimplantation genetic testing for monogenic disorders (PGT‐M), which led to a successful pregnancy.

## 2 SUBJECTS AND METHODS

### 2.1 Sample preparation

The present study was approved by the Ethics Committee of Affiliated Shenzhen Maternity and Child Healthcare Hospital, Southern Medical University. The couple provided their written informed consent. Tissue from the aborted fetus and peripheral blood samples from the parents were collected for further analysis.

### 2.2 Low‐coverage whole‐genome sequencing

Low‐coverage whole‐genome sequencing (LC‐WGS) was employed to identify chromosomal abnormalities, which was performed as previously described (Geng et al., 2019). Any deletion or duplication larger than 0.5 megabase (Mb), as well as any aneuploidy, were reported.

### 2.3 Trio‐based whole exome sequencing

Trio‐based whole exome sequencing (trio‐WES) was performed to investigate the potential pathogenic variants associated with MKS. Total genomic DNA was extracted from the tissue of the aborted fetus and peripheral blood samples of the parents. DNA fragments were hybridized and captured by the Berry Custom Design V2 kit (Berry Genomics, China) according to manufacturer's protocol. Then the sequencing library was sequenced on the NovaSeq 6000 platform (Illumina, Inc, USA) with 150 bp paired‐end reads. The sequencing reads were aligned to the human reference genome (hg19/GRCh37) with BWA (v0.7.17).

### 2.4 In vitro fertilization, PGT‐M, and prenatal diagnosis

Detailed methods description of in vitro fertilization, PGT‐M, and prenatal diagnosis is presented in Data S1.

## 3 RESULTS

### 3.1 Clinical case presentation

A 35‐year‐old Chinese woman was referred for clinical genetics consultation after multiple congenital anomalies were detected on a fetal ultrasonography during the 12th gestational week after in vitro fertilization (IVF) and embryo transfer. Multiple congenital anomalies were found through antenatal ultrasound scan includes occipital encephalocele, omphalocele, bilateral polycystic kidneys, enlarged nuchal translucency, postaxial polydactyly, and oligohydramnios. The abnormal pregnancy was subsequently terminated. Prior to this adverse pregnancy, the woman had experienced two missed abortions in 2014 and 2017, respectively, and a termination of pregnancy due to the similar congenital anomalies detected by fetal ultrasonography during the 24th week of gestation in 2015. Consanguinity was denied, and family history was negative for severe genetic disorders. Given these findings, MKS was suspected in the proband. To confirm the diagnosis, genetic analysis was performed.

### 3.2 Genetic analysis results

**FIGURE 1:**
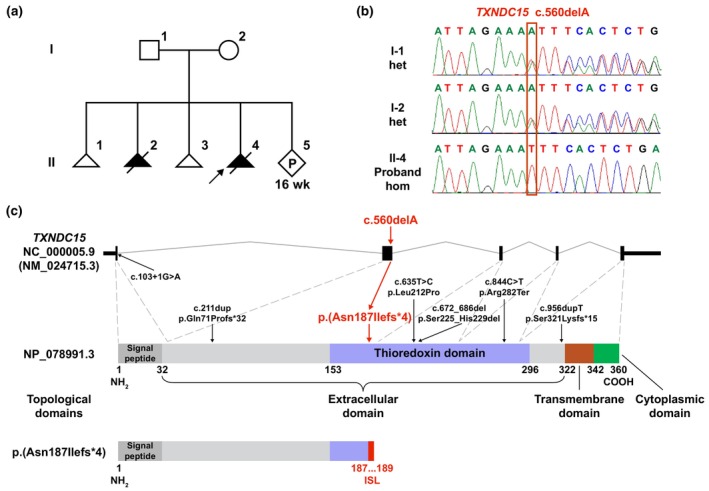
Genetic analysis of the family. (a) Pedigree of the non‐consanguineous Chinese family with a novel *TXNDC15* variant. The parents (I‐1 and I‐2) are the unaffected heterozygous carriers of the variant (c.560delA). While the proband (II‐4) is a terminated pregnancy due to multiple congenital anomalies found through antenatal ultrasound scan, which is caused by the homozygous *TXNDC15* variant. II‐2 is also a terminated pregnancy due to similar multiple congenital anomalies on antenatal ultrasound scan in 2015. II‐1 and II‐3 are the two missed abortions in 2014 and 2017, respectively. (b) Sanger sequencing of the family members I‐1, I‐2, and II‐4 (the proband). The red rectangle indicates the variant site (c.560delA). (c) Schematic representation of the *TXNDC15* gene (NM_024715.3, first panel) and its protein structure retrieved from the UniProt database (Q96J42, second panel). The relative positions of the variants that have been reported are illustrated by pointing to the gene structure and the protein structure, respectively. The variant site reported here is marked red. Functional domains of *TXNDC15* protein are depicted in the protein structure panel, while its topological domains are shown under the panel. The last panel shows the theoretical protein structure of the *TXNDC15* variant reported here, although which should not be synthesized due to nonsense‐mediated decay of its mRNA.

Considering the mother experienced recurrent miscarriages, LC‐WGS was performed to rule out a chromosomal anomaly and confirmed that the aborted fetus did not possess any known pathogenic deletions or duplications. The trio‐WES analysis identified a novel homozygous variant (NM_024715.3: c.560delA, p.(Asn187Ilefs*4)) in the *TXNDC15* gene of the proband, which was inherited from both parents (Figure 1a,b). This variant was confirmed by Sanger sequencing and has not been seen in the general population databases (1000 genome project database [https://www.internationalgenome.org/], ExAC database [https://exac.broadinstitute.org/], and gnomAD [http://www.gnomad‐sg.org/]). The frameshift variant results in a premature stop codon which is predicted to cause its mRNA subjected to nonsense‐mediated decay (NMD) (Lindeboom et al., 2019). Thus, the predicted protein variant should not be synthesized due to NMD. The variant was classified as “pathogenic” (PVS1 + PM2_supporting + PP4) according to the variant interpretation guideline of the American College of Medical Genetics and Genomics (Richards et al., 2015).

### 3.3 Outcome of PGT

As the pathogenic variant was identified, the couple chose to undergo PGT‐M in order to avoid transmitting the variant to their offspring. In total, three blastocysts were obtained and biopsied. For each biopsy sample, 60‐60‐55 informative polymorphic SNP markers located upstream and downstream of the *TXNDC15* gene were available to evaluate the haplotype, with 30 of these markers shown in Figure S1. Sanger sequencing confirmed the SNP‐based haplotyping results. The PGT results and clinical outcomes of the three biopsied embryos are summarized in Table 1 (Figure S1).

**TABLE 1:** Clinical outcomes of the three embryos.

Embryo	Biopsy time	Grading^a^	Sanger sequencing	SNP haplotyping^b^	CNV	Clinical outcome
E1	D5	4AB	c.560delA/c.560delA	F‐Hap A/M‐Hap A	46,XN	Abandoned
E2	D6	4BB	c.560delA/N	F‐Hap A/M‐Hap B	46,XN	Frozen
E3	D6	3BB	Wild‐type	F‐Hap B/M‐Hap B	46,XN	Transferred

^a^ The embryo quality was assessed following the Gardner grading system.

^b^ F‐Hap A: haplotype A of the father, c.560delA; F‐Hap B: haplotype B of the father, wild‐type; M‐Hap A: haplotype A of the mother, c.560delA; M‐Hap B: haplotype B of the mother, wild‐type.

Finally, the diploid embryo E3 which do not harbor the *TXNDC15* variant was selected for transfer, resulting in a successful pregnancy confirmed by human chorionic gonadotropin and ultrasound examination. At 16th gestational week, Sanger sequencing of amniotic fluid DNA confirmed that the fetus carried the wild‐type *TXNDC15* gene (Figure S2). Additionally, the chromosomal microarray analysis result showed that no CNV larger than 100 kb was identified in the fetus. Prenatal ultrasound examination showed an ongoing pregnancy with normal fetal development.

## 4 DISCUSSION

Thioredoxin domain containing 15 (*TXNDC15*) gene, located in 5q31.1, encodes a member of the thioredoxin superfamily which intricate in disulfide isomerase activity. *TXNDC15* was first identified as a causative gene of MKS14 in three independent families which share the cardinal features of MKS with different variants (c.672_686del, c.103+1G>A, c.956dup) in 2016 (Shaheen et al., 2016). Since then, only two other novel cases have been reported: compound heterozygous variants c.(211dup/635T>C) in a 12‐week‐old fetus from an Estonian family and a homozygous variant c.844C>T in a 14‐week‐old fetus from an Indian family (Radhakrishnan et al., 2019; Ridnõi et al., 2019). We summarized the *TXNDC15* variants and corresponding patient clinical data previous reports as well as the current case in Table 2 and Figure 1c. Half of the cases (3/6) were found in consanguineous families. While most patients present with typical symptoms such as enlarged polycystic kidneys, occipital encephalocele, and postaxial polydactyly, other clinical phenotypes including reproductive system anomalies, central nervous system anomalies, hepatic fibrosis, and others may also occur. Previous research suggests that pathogenic *TXNDC15* variants prevent correct localization of the TMEM67 ciliary receptor to the transition zone, which caused a decreased number of ciliated cells and abnormal ciliary morphology (Shaheen et al., 2016). Breslow et al. (2018) found a clear defect in Hh signaling in mouse *Txndc15*‐knockout cells. Despite these findings, the specific molecular mechanism underlying *TXNDC15* variants causing MKS requires further investigation.

**TABLE 2:** Clinical and molecular findings of *TXNDC15* variants in five previously reported families as well as in this study.

Family	*TXNDC15* variant (NM_024715.3)	Ethnic origin	Age	Phenotype	Consanguineous	Family history
1 (Shaheen et al., 2016)	Homozygous c.672_686del p.(Ser225_His229del)	Saudi	Stillbirth	Prenatal findings: suspicious for MKS. After delivery: polydactyly, enlarged polycystic kidneys, and occipital encephalocele	Yes	+
2 (Shaheen et al., 2016)	Homozygous c.103+1G>A	Saudi	Fetus	Prenatal findings: encephalocele, enlarged polycystic kidneys and polydactyly, oligohydramnios. Postnatal examination: cyanosis and poor respiratory, normal growth parameters, bilateral microphthalmia, posterior encephalocele, low set ears, microretrognathia, abnormally shaped chest with widely spaced underdeveloped nipples, protuberant abdomen with bilaterally palpable kidneys, ambiguous genitalia with absent phallus and absent vaginal opening, postaxial polydactyly, and syndactyly	Yes	−
3 (Shaheen et al., 2016)	Homozygous c.956dupT p.(Ser321Lysfs*15)	Pakistani	Fetus	Occipital encephalocele, polycystic kidneys, polydactyly, low set ears, micrognathia, hypertelorism, bilateral talipes, and absent uterus	Yes	+
4 (Ridnõi et al., 2019)	Compound heterozygous c.211dup/c.635T>C p.(Gln71Profs*32)/p.(Leu212Pro)	Estonian	Fetus (13 weeks)	Prenatal findings: enlarged nuchal translucency, bilateral polycystic kidneys, occipital encephalocele, postaxial polydactyly. Autopsy result: occipital encephalocele, bilateral enlarged polycystic kidneys with total kidney, bilateral polydactyly (six fingers and seven toes). histological study confirmed polycystic dysplastic kidneys	No	−
5 (Radhakrishnan et al., 2019)	Homozygous c.844C>T p.(Arg282*)	Indian	Fetus (14 weeks)	Autopsy result: bilateral polycystic kidneys, holoprosencephaly, occipital encephalocele, bilateral polydactyly, micro‐retrognathia, anteverted nares, widely placed eyes, short neck, bowed, long bones of leg and single cardiac ventricle with common atrioventricular opening, cystic lesions in the kidneys, under‐mineralized cranial vault, bilateral bent tibia, and fibula	No	−
6^a^	Homozygous c.560delA p.(Asn187Ilefs*4)	Chinese	Fetus (12 weeks)	Prenatal findings: occipital encephalocele, omphalocele, bilateral polycystic kidneys, enlarged nuchal translucency, postaxial polydactyly, oligohydramnios	No	−

Abbreviation: MKS, Meckel–Gruber syndrome.

^a^ Family 6 is the case reported in this study.

As described in the results section, the woman who came to us had previously experienced four adverse pregnancies. In 2015, the couple had a fetus suspected of having MKS due to characteristic symptoms at 24 weeks of gestation. The couple underwent a targeted‐gene sequencing panel of 28 known MKS‐related genes at that time, although *TXNDC15*, which was later identified as an MKS‐related gene in 2016, was not included in the test. The couple exhibited normal G‐banded karyotypes at 550‐band resolution, indicating no large‐scale chromosomal anomalies (data not shown). As the test results were negative, they attempted natural conception once again in 2017 but suffered another missed abortion. In 2021, they came to us seeking routine IVF treatment and got pregnant. However, multiple congenital anomalies were detected again on fetal ultrasonography at 12 weeks of gestation, as mentioned earlier. To this end, trio‐WES was finally used to identify the potential causal variants. This case clearly shows that making clinical decision based on gene sequencing panel years ago can lead to missed diagnosis. By reflecting on the diagnostic process, clinicians should be aware of the limitations under gene panel sequencing that only several dozens of known causal genes of suspected disease are targeted, which can miss the unknown disease‐causing gene. In fact, the *TXNDC15* variant c.560delA of the proband was initially classified as “uncertain significance (VUS)” since its clinical validity curation had not been resolved. We evaluated the strength of evidence supporting or refuting a claim that variation in *TXNDC15* gene causes MKS by analyzing previous literature and applying the ClinGen clinical validity framework (Strande et al., 2017). Finally, gene–disease relationships of the *TXNDC15* were classified as “strong,” allowing us to re‐classify the variant as “pathogenic.” Then, the couple decided to receive PGT‐M for the *TXNDC15* variant and achieved an ongoing pregnancy. Therefore, periodically re‐analyzing and re‐interpreting negative test reports, continuously updating disease genes and pathogenic variants, and focusing particularly on follow‐up of high‐risk couples might all contribute to the ending of diagnostic odyssey. This case emphasizes the crucial role of accurate genetic counseling and that variant classifications may be subjected to change with the availability of new information.

In summary, we describe a case of a fetus affected by MKS from a non‐consanguineous Chinese family caused by a novel homozygous variant c.560delA in the *TXNDC15* gene. This is the first report of a pathogenic *TXNDC15* variant in the Chinese population and the first PGT case of *TXNDC15*‐related MKS worldwide. Our case expands the variant spectrum of the *TXNDC15* gene, which further aids in both the molecular diagnosis and genetic counseling of MKS.

## AUTHOR CONTRIBUTIONS

*Devised the idea for this study*: Xuemei Li, Huiling Xu, and Ningjie Yang. *Discussed the results and contributed to the final manuscript*: Huiling Xu and Jiajie Pu. *Obtained patient materials and did clinical operation of PGT*: Chanlin Han and Xuemei Li. *Performed PGT*: Zhengzhong Wu. All authors read and approved the final manuscript.

## FUNDING INFORMATION

The study was carried out with the funding support of the Key Medical Subject of Shenzhen (2020–2024) (SZXK031) and the Research Program of Science, Technology and Innovation Commission of Shenzhen Municipality (2020231557).

## CONFLICT OF INTEREST STATEMENT

The authors declare no competing interests.

## ETHICS STATEMENT

The present study was approved by the Ethics Committee of Shenzhen Maternity and Child Healthcare Hospital of Southern Medical University (No. 2021‐86). The couple in this case provided written informed consent.

## Supporting information


Data S1.

## Data Availability

The datasets generated and analyzed during the current study are not publicly available due to individual privacy or ethical restrictions but are available from the corresponding author on reasonable request.
