# Supplementary material for: First preimplantation genetic testing case of Meckel syndrome with a novel homozygous TXNDC15 variant in a non‐consanguineous Chinese family
Source: Mol Genet Genomic Med. 2023 Dec 11;12(1):e2340. doi: 10.1002/mgg3.2340 (PMC10767674; doi:10.1002/mgg3.2340)
Supplement: Supplementary file 1 — Data S1. [file MGG3-12-e2340-s001.pdf]

Supplementary Information for
"First preimplantation genetic testing case of Meckel syndrome with a novel homozygous *TXNDC15* variant in a non-consanguineous Chinese family"

Supplementary Methods

In vitro fertilization and preimplantation genetic testing (PGT)

Oocyte pickup (OPU) was undertaken after controlled ovarian stimulations administrated. Due to concern for genetic contamination from other spermatozoa, intracytoplasmic sperm injection (ICSI) was chosen as the fertilization technique. Then all of blastocysts were subjected to trophectoderm cell biopsy on day 5 or day 6 via laser. Approximately 5–10 cells were extracted from the trophectoderm (TE) of each blastocyst and subjected to whole-genome amplification (WGA). Biopsied TE cells from each embryo were applied following the standard protocol of WGA method of multiple annealing and looping based amplification cycles (MALBAC) (Yikon Genomics) (Zong et al., 2012). WGA products of each embryo were subjected to Sanger sequencing to identify variant sites directly. To prevent misdiagnosis due to allele drop-out, haplotyping analysis was conducted through SNP markers with sequencing depth ≥100× within the 1Mb region flanking the target gene (in our case, *TXNDC15*) via targeted capture sequencing. The method has been described in previous publication (He et al., 2022; Masset et al., 2022). The peripheral blood DNA information of the couple and the proband was used to construct the family SNP haplotypes. Then informative SNP markers were selected to identify the disease carrying haplotype phase in each embryo. Besides the detection of mutation, CNV analysis was also carried out on all embryos to prevent embryonic abortion, death or other problems may be caused by embryonic chromosomal abnormalities. Any deletion or duplication more than 4Mb and mosaicism more than 30% in each embryo were reported.

Prenatal diagnosis

Clinical pregnancy was defined as the presence of a fetal heartbeat by sonography 28 days after frozen embryo transfer (FET). Prenatal molecular diagnosis was performed through amniocentesis at 16th gestational week to confirm the diagnosis of PGT. The fetal genotype was confirmed by Sanger sequencing. Aneuploidy, microdeletion and microduplication larger than 100kb were detected through CMA using CytoScan 750K (Afymetrix, Inc, USA).

References

He, B., Wang, L., Wu, Q., Wang, X., Ji, X., Shi, W., Shi, J., Qiang, R., & Zhen, S. (2022). Clinical application of NGS-based SNP haplotyping for PGT-M of methylmalonic acidemia. *Systems Biology in Reproductive Medicine*, *68*(1), 80–88. https://doi.org/10.1080/19396368.2021.2005718

Masset, H., Ding, J., Dimitriadou, E., Debrock, S., Tšuiko, O., Smits, K., Peeraer, K., Voet, T., Zamani Esteki, M., & Vermeesch, J. R. (2022). Single-cell genome-wide concurrent haplotyping and copy-number profiling through genotyping-by-sequencing. *Nucleic Acids Research*, *50*(11), e63. https://doi.org/10.1093/nar/gkac134

Zong, C., Lu, S., Chapman, A. R., & Xie, X. S. (2012). Genome-wide detection of single-nucleotide and copy-number variations of a single human cell. *Science*, *338*(6114), 1622–1626. https://doi.org/10.1126/science.1229164

Supplementary Figures

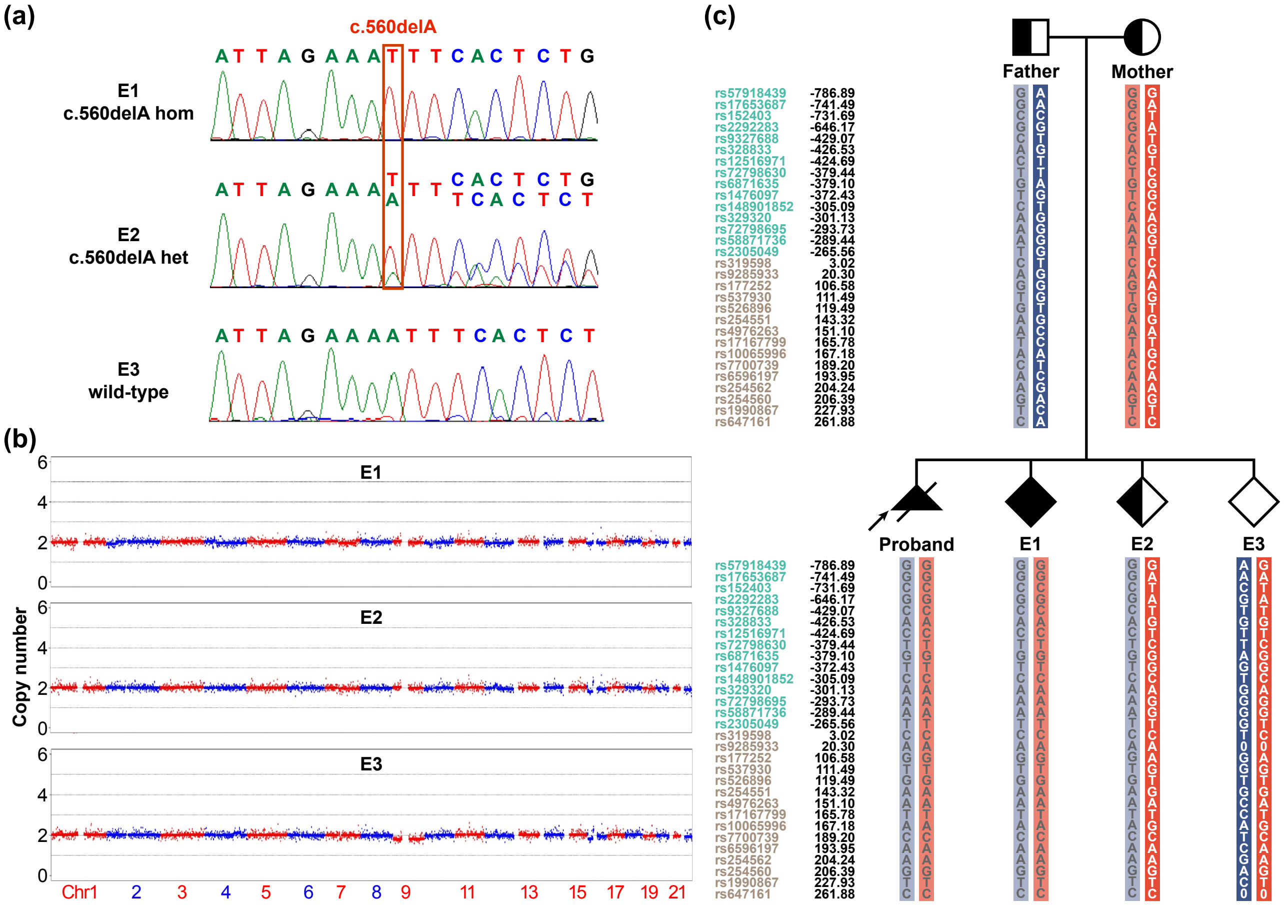

FIGURE S1 PGT results of the three embryos. (a) Sanger sequencing results of biopsied blastocysts. The loci of the variant c.560delA are marked in red box. Compared with the sequence of wild-type *TXNDC15*, embryo 1 (E1) carried biallelic *TXDNC15* variants, embryo 2 (E2) carried the heterozygous variant while embryo 3 (E3) did not carry the *TXNDC15* variant (c.560delA). (b) Copy number variation (CNV) results of embryos. Compared with the hg19 reference genome through CNV analysis, the sketch maps shown that all samples were diploid. (c) Diagram showing the results of haplotype analyses of the father, mother, proband and 3 blastocysts (E1–3). E1 carried the affected haplotypes from the couple. E2 inherited an affected haplotype (alleles in gray font on the dark blue bar) from the father, whereas E3 inherited the normal haplotypes from the parents. Cyan and light brown represents SNP markers located upstream and downstream of the *TXNDC15* gene, separately.

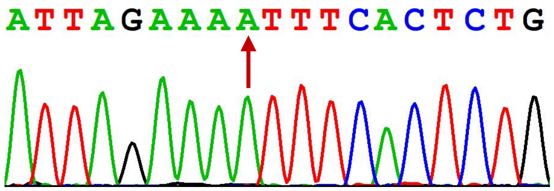

FIGURE S2 Sanger sequencing of *TXNDC15* for the fetus at 16 gestational weeks developed from the embryo E3 confirms the PGT results. Red arrow indicates the variant site in *TXNDC15* reported here.
